# To test or not to test for body fluids: integration of body fluid identification and direct PCR in one workflow

**DOI:** 10.1093/fsr/owaf025

**Published:** 2025-09-13

**Authors:** Francisco Medina-Paz, Gabriela Roca, Christian Stadler, Santina Castriciano, Sara C Zapico

**Affiliations:** Department of Chemistry and Environmental Sciences, New Jersey Institute of Technology, Newark, USA; SERATEC mbH, Göttingen, Germany; SERATEC mbH, Göttingen, Germany; Copan Italia Spa, Brescia, Italy; Department of Chemistry and Environmental Sciences, New Jersey Institute of Technology, Newark, USA; Anthropology Department and Laboratories of Analytical Biology, National Museum of Natural History, Smithsonian Institution, Washington, DC, USA

**Keywords:** forensic sciences, forensic genetics, body fluids, direct PCR, STR profiling, immunochromatographic tests

## Abstract

Frequently at crime scenes, it is possible to find lesser amounts of biological material, which prevents performing all the analyses to make a full identification of the evidence: body fluid identification, DNA extraction, human DNA quantitation, and short tandem repeats (STR) profiling. In these situations, DNA profiling is chosen over body fluid identification. Nowadays, the current advancements in forensic genetics, such as the development of different swab materials and direct polymerase chain reaction (PCR) amplification, allow us to skip the steps of DNA extraction and quantitation, avoiding losing important amounts of genetic material and original evidence. However, DNA profiling is as important as body fluid identification in certain cases. The present study assessed the efficiency of integrating body fluid identification by immunochromatographic tests and genetic profiling into a single workflow using microFLOQ® swabs and evaluating different approaches in bloodstain samples. The findings from this research indicated that the microFLOQ can be used both for sampling directly from the source and for subsampling from swabs of different materials, followed by direct PCR to get good-quality STR profiles, in this case allowing to extract the maximum information from a “unique” source of evidence before destruction, as in body fluid and genetic identification. Future research can expand these results to other body fluids (i.e., semen and saliva) and mixtures.

**Key points**
 

- The present work showed that the integration of body fluid identification by immunochromatographic tests and genetic profiling by STR analysis into a single workflow is feasible.
- Three different strategies for integrating body fluid identification and genetic profile into one single workflow were assessed with different results.
- No clear correlation was found between hemoglobin concentration and the quality of the STR profiles.
- A viable solution for low-quantity DNA casework scenarios may be using microFLOQ for subsampling from regular cotton or nylon-flocked (4N6FLOQ) swabs.

## Introduction

Biological evidence at crime scenes could be significantly degraded due to environmental factors such as temperature, humidity, radiation, etc. [1]. In addition to the degradation, it is not rare to find only lesser amounts of biological evidence at crime scenes. This situation limits the implementation of complete protocols for the identification of the evidence, from body fluid identification to DNA analysis. The standard workflow of forensic identification of biological evidence typically begins with the assessment of the provenance of the sample through body fluid identification [2]. Once it is presumptively identified, the remaining sample of biological evidence is then used for DNA analysis by isolating DNA from the evidence, quantifying the human genetic material, and generating a genetic profile, aiming to compare with a reference sample from a possible perpetrator or crime victim [3]. The whole process of DNA identification and analysis involves the extraction and use of a considerable amount of good-quality genetic material, hence the goal is always to conserve and reduce the consumption of evidence to the minimum necessary for testing [4].

Determination of the origin of the body fluids found at a crime scene can provide valuable information to perform crime scene reconstruction. Numerous types of body fluid identification methods have been developed, such as alternative light source (ALS) detection, chemical tests, enzymatical tests, immunological tests, and more recently, proteomics [5]. However, conventional body fluid identification approaches are mostly presumptive, and their application is restricted to only one body fluid at a time [6]; this restriction does not align to minimize the consumption of evidence. For the other part, although proteomics represents a promising emerging alternative for the identification of more than one body fluid at a time, current techniques still require the characterization and validation of new and highly reproducible protein markers; not to mention the development of standardized protocols optimized for small sample amounts as well as the creation of databases for analysis [7].

Immunochromatographic tests, widely used for body fluid identification, are conducted after a potential fluid stain is found and before DNA testing is carried out [8]. These tests rely on the binding of antigens present in a sample of the potential body fluid extracted from the evidence and diluted in a volume of buffer provided by the manufacturer, to the immobilized antibodies of the tests [9]. If DNA profiling follows the body fluid identification testing, the remaining sample or an additional portion of the evidence sample is used while the test result line strip is discarded [4]. Some studies have shown that DNA and genetic profiles can be obtained from immunochromatographic test result line strips for peripheral blood, menstrual blood, saliva, and semen [4,10,11], increasing the efficiency of testing by using one single extraction for both body fluid identification and DNA profiling. However, this is not a common procedure in forensic labs [12]. Therefore, there is a need for an improved workflow that increases efficiency in terms of generating quality STR profiles while reducing sample consumption.

Regarding the collection method, cotton swabs have been traditionally used for the retrieval of body fluid samples at crime scenes [13]. Nevertheless, following questions raised some years ago about their design and limitations in terms of their effectiveness in recovering and subsequently releasing the extracted sample (20%–76% losses), options such as nylon-flocked swabs have been developed [14]. In contrast to regular cotton swabs, 4N6FLOQSwabs® (Copan Italia, Brescia, Italy) consist of short nylon fibres arranged perpendicularly at the tip of an applicator shaft [15].

One of the most recent designs of nylon-flocked swabs is the microFLOQ® Direct Swab (Copan Italia). According to the manufacturer, the arrangement of the fibre of this swab is the same as 4N6FLOQSwabs®, but they differ in that the latter are treated with a lysing agent for direct amplification, eliminating the need for DNA isolation and quantification [16]. MicroFLOQ swab offers two main advantages: (i) Due to the small dimension of its head, only a minimal portion of the sample is collected, consuming far less sample than traditional swabs and (ii) direct DNA amplification can be easily performed by cutting the head of the swab, eliminating the genetic material isolation and quantitation steps from the sample processing workflow. Therefore, this study proposes the use of microFLOQ swabs as a core part of the development of a strategy for the integration of body fluid identification processes and the generation of genetic profiles.

The present study evaluated the feasibility of an integrated workflow for body fluid identification and genetic profiling. Three different workflows were performed using different swab materials and different strategies for reducing sample consumption while optimizing genetic profiling through STR sequencing. Our findings demonstrated that it is possible to integrate body fluid identification and genetic profiling into a single workflow using microFLOQ swabs and sampling either directly from the source (in this case blood spots) or swabs of different materials, followed by direct PCR to obtain good quality STR profiles.

## Materials and methods

### General experimental design

**Figure 1:**
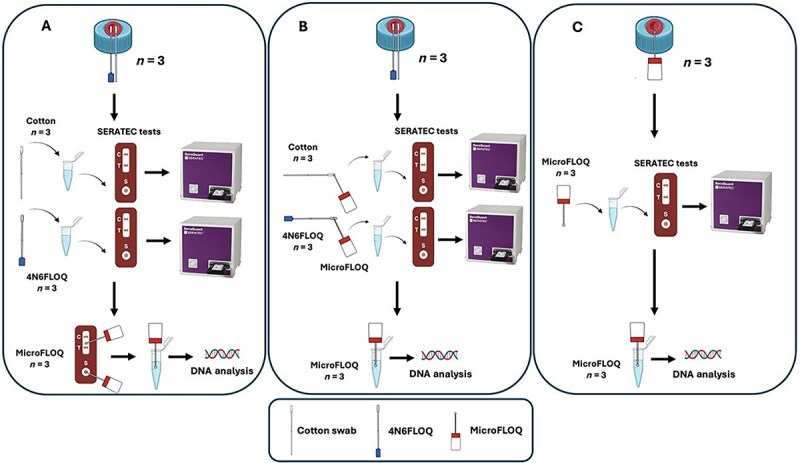
Schematic representation of the experimental design showing the different methodologies divided into three experimental groups. (A) Experiment 1: microFLOQ swabs subsampling from SERATEC cards (*n* = 24). (B) Experiment 2: swab subsampling using microFLOQ swabs (*n* = 6). (C) Experiment 3: microFLOQ swabs direct sampling (*n* = 6).

The New Jersey Institute of Technology Institutional Review Board (IRB) approved the procedures related to human body fluid experimentation (protocol number: 2110013076). Blood from an African American woman was purchased from the American Blood Bank Corporation (Miami, FL, USA). The general experimental setup consisted of depositing 20 μL of human (female) blood on eighteen 50 mL Falcon® tubes’ caps. Falcon tubes’ caps are made of polyethylene (plastic) [17], one of the most common surfaces for body fluid sampling [18]. The samples were left under a biosafety cabinet at room temperature (20°C), between 30% and 50% humidity, and under a constant airflow at 973 m^3^/h, for 48 h. After 48 h, the samples were divided into three different experiments as shown in Figure 1.

### Experiment 1: microFLOQ swabs subsampling from SERATEC cards

Two different swab types were used: cotton swabs (cotton-tipped applicators sterile, wood shaft; SARSTEDT, Nümbrecht, Germany), and Copan 4N6FLOQSwabs regular-size tip (Figure 1A). Both swab types were moistened in the buffer provided with the SERATEC® HemDirect Hemoglobin Tests [19]. Once moisturized, samples were collected rubbing the swab on the plastic cap with the dry bloodstain in a circular motion for ~30 s. Three replicates were conducted for each swab type. Then, swabs were incubated under agitation in 300 μL of SERATEC buffer for 10 min as recommended by the test manufacturer. Afterward, three drops (~120 μL) of the resulting solution were added to the immunochromatographic tests, SERATEC HemDirect, using the plastic pipette provided with each test, and the results were recorded. The volume was added using the plastic pipette provided by the manufacturer to stick as closely as possible to a real scenario of test use.

While still wet, subsampling from the immunochromatographic tests was performed using microFLOQ swabs, previously moisturized using MiliQ water, touching: (i) on the test’s sample pad and (ii) on the test’s result line (Figure 1A). Two additional subsamples from the same parts of the tests were taken when they were completely dry.

Finally, the head of the microFLOQ swab was used for direct PCR amplification by snapping off the tip of the microFLOQ into PCR strip tubes and amplified using the Promega PowerPlex® Fusion 6C System (Promega Corporation, Madison, WI, USA). The volume of the sample solution was replaced with molecular-grade water and the PCR master mix was added directly into the tubes.

### Experiment 2: swab subsampling using microFLOQ swabs

Regular cotton swabs and nylon-flocked swabs (now referred to as 4N6FLOQ) were moistened in the buffer provided with SERATEC HemDirect (Figure 1B). Then, the swab sampling was performed by rubbing the swab on the plastic cap with the dry bloodstain in a circular motion for ~30 s. Three replicates were carried out for each swab type.

Before incubating the swabs on the SERATEC buffer, water moisturized microFLOQ swabs were used for subsampling from both the regular cotton and 4N6FLOQ by touching their tips. After sampling with the microFLOQ swabs, cotton and 4N6FLOQ swabs were incubated under agitation in 300 μL of SERATEC buffer for 10 min. Three drops of the resulting solution were added to the immunochromatographic tests, and the results were recorded. Finally, the head of the microFLOQ swab was used for direct PCR amplification according to the manufacturer’s instructions.

### Experiment 3: microFLOQ swabs direct sampling

MicroFLOQ swabs, moisturized with SERATEC buffer, were used to retrieve the blood samples directly from the 50 mL Falcon tubes’ caps (Figure 1C). Sampling was performed by touching the sample numerous times at different points of the dry bloodstain. The samples adhered to the swabs were placed in Eppendorf tubes with different volumes of SERATEC buffer (60 and 120 μL) to dilute the blood sample. The diluted samples in the tubes were incubated under agitation for 10 min. Then, the whole volume of the resulting solution was added to the immunochromatographic tests sample pad, and the results were recorded. Finally, the head of the microFLOQ swab with the remnants of the initially collected bloodstain sample was used for direct PCR amplification according to the manufacturer’s instructions.

### Hemoglobin protein quantification

The SERATEC SeraQuant (SERATEC, Goettingen, Germany) was used to assess the results of the immunochromatographic tests by converting band intensity to hemoglobin (Hb) concentration in ng/mL units. The SERATEC SeraQuant is an instrument for the quantitative analysis of the SERATEC test cards for the detection of human body fluid markers. The calibration curve for Hb protein was performed according to the manufacturer’s instructions.

### Nuclear DNA profiling

The Promega PowerPlex Fusion 6C System (Promega Corporation) was used to amplify and characterize 23 autosomal STRs, three Y-STRs, and the Amelogenin (*AMEL*) gene from the microFLOQ swabs, following the manufacturer’s direct PCR protocol. For all experiments, the direct STRs amplification was performed by cutting the tip of the microFLOQ swabs and placing it directly inside of the tube where the reagents for the PCR reaction were already added. After the amplification was performed, fragment analysis was carried out on SeqStudio Genetic Analyzer (Thermo Fisher Scientific, Waltham, MA, USA). The fragment analysis was performed under the following parameters: 7 s injection time; 1 200 volts injection voltage; 1 440 s run time; and 9 000 volts run voltage. DNA profiling was achieved through the Microsatellite Analysis software on Thermo Fisher Cloud (https://www.thermofisher.com/rs/en/home/digital-science/thermo-fisher-connect.html, accessed on 20 March 2024). A threshold of 150 relative fluorescence units (RFUs) for peak detection/analysis was used. Two different parameters were calculated to assess the quality of the DNA as previously described [20]: total peak height (TPH): the total sum of the height of the peaks in a profile; and peak height ratio (PHR): the height of the smaller peak in a heterozygote pair divided by the height of the larger peak.

### Statistical analysis

Statistical analyses were conducted using custom R scripts. All data were analysed using R version 4.2.2 [21] and R Studio version 2023.06.0 + 421 [22]. Non-parametric Kruskal–Wallis and Dunn’s *post hoc* tests were performed to evaluate different swab materials for initial sampling.

## Results

### Hemoglobin quantification

**Figure 2:**
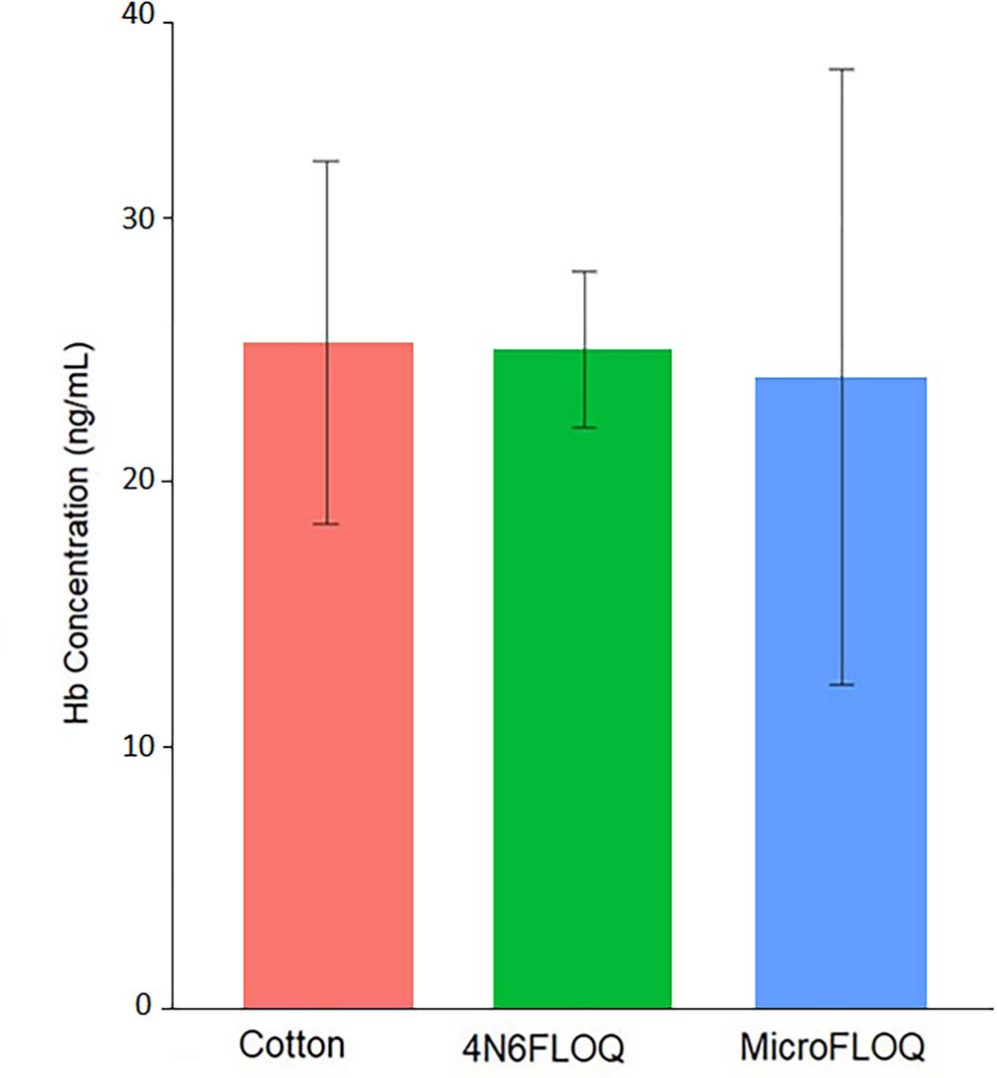
Hemoglobin (Hb) concentration (ng/mL) of blood using regular cotton, 4N6FLOQ, and microFLOQ swabs. Hb concentration was measured using SERATEC SeraQuant (Kruskal-Wallis test and Dunn’s *post hoc* test, *P* < 0.05, *n* = 3).

Results of the Hb concentration from the SERATEC cards using the SERATEC SeraQuant (Figure 2) showed no statistically significant difference (*P* = 0.54) among the materials of the swabs used for the initial sampling: cotton, 4N6FLOQ, and microFLOQ.

### Experiment 1: microFLOQ swabs subsampling from SERATEC cards

From 24 observations where all the Hb tests were positive, not fully reliable STR profiles could be recovered either from the wet or the dry SERATEC cards when subsampling using microFLOQ swabs.

As reported in Table 1, all the replicates from bloodstains sampled using regular cotton swabs showed STR profiles not consistent with donor’s reference profile, both when subsampling from wet SERATEC cards’ result line strip or well sample pad. Regarding the dry SERATEC cards, one out of the three replicates presented STR profiles that did not correspond to the donor’s profile neither to the ones of the researchers and might correspond to environmental contamination [23], both for the result line strip and the sample pad (replicate 3). For the two remaining replicates of the dry SERATEC cards, contaminated STR profiles were observed for the replicate test line strip of replicate 1 and for the sample pad of replicate 2, while non-consistent STR profiles were observed for sample pad of replicate 1 and for result line strip in replicate 2.

**Table 1:** Experiment 1 samples description, results, and observations. Yielded profile = observed/expected number of alleles

Sample ID	Swab material	Sample description	Replicate	Result	Yielded profile	Observations
ce11ws	Cotton	Wet result line strip	1	Non-consistent profile	–	STR profile non consistent with donor’s reference profile
ce11ww	Cotton	Wet sample pad	1	Non-consistent profile	–	STR profile non consistent with donor’s reference profile
ce12ws	Cotton	Wet result line strip	2	Non-consistent profile	–	STR profile non consistent with donor’s reference profile
ce12ww	Cotton	Wet sample pad	2	Non-consistent profile	–	STR profile non consistent with donor’s reference profile
ce13ws	Cotton	Wet result line strip	3	Non-consistent profile	–	STR profile non consistent with donor’s reference profile
ce13ww	Cotton	Wet sample pad	3	Non-consistent profile	–	STR profile non consistent with donor’s reference profile
ce11ds	Cotton	Dry result line strip	1	Contamination	26/48	*AMEL* Y and Y-STRs amplified
ce11dw	Cotton	Dry sample pad	1	Non-consistent profile	–	STR profile non consistent with donor’s reference profile
ce12ds	Cotton	Dry result line strip	2	Non-consistent profile	–	STR profile non consistent with donor’s reference profile
ce12dw	Cotton	Dry sample pad	2	Contamination	40/48	*AMEL* Y and Y-STRs amplified
ce13ds	Cotton	Dry result line strip	3	Contamination	40/48	*AMEL* Y and Y-STRs amplified
ce13dw	Cotton	Dry sample pad	3	Contamination	46/48	*AMEL* Y and Y-STRs amplified
411ws	4N6FLOQ	Wet result line strip	1	Non-consistent profile	–	STR profile non consistent with donor’s reference profile
411ww	4N6FLOQ	Wet sample pad	1	Negative	0/48	None
412ws	4N6FLOQ	Wet result line strip	2	Contamination	8/48	Very low height, out of limit
412ww	4N6FLOQ	Wet sample pad	2	Contamination	6/48	Very low height, out of limit, *AMEL* Y
413ws	4N6FLOQ	Wet result line strip	3	Contamination	14/48	Very low height, out of limit, *AMEL* Y
413ww	4N6FLOQ	Wet sample pad	3	Contamination	2/48	Very low height, out of ladder
411ds	4N6FLOQ	Dry result line strip	1	Contamination	30/48	*AMEL* Y and Y-STRs amplified
411dw	4N6FLOQ	Dry sample pad	1	Contamination	24/48	*AMEL* Y and Y-STRs amplified
412ds	4N6FLOQ	Dry result line strip	2	Contamination	34/48	*AMEL* Y and Y-STRs amplified
412dw	4N6FLOQ	Dry sample pad	2	Contamination	34/48	*AMEL* Y and Y-STRs amplified
413ds	4N6FLOQ	Dry result line strip	3	Contamination	32/48	*AMEL* Y and Y-STRs amplified
413dw	4N6FLOQ	Dry sample pad	3	Contamination	34/48	*AMEL* Y and Y-STRs amplified

**Figure 3:**
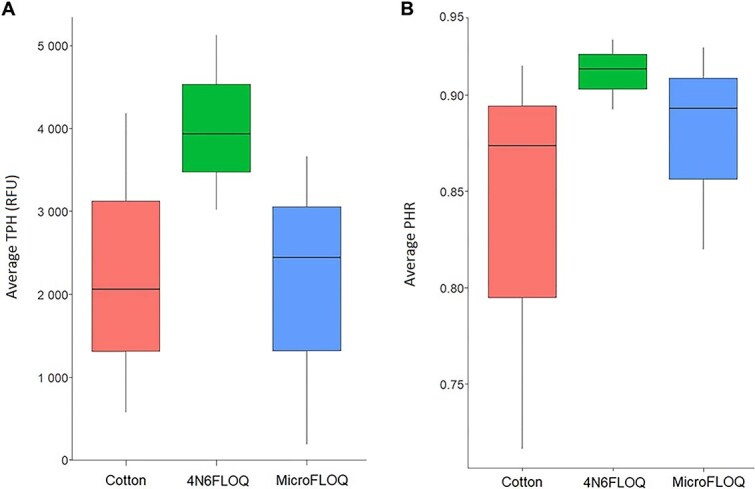
(A) Average of total peak height (TPH) expressed as relative fluorescence units (RFUs). (B) Average peak height ratio (PHR) expressed as a ratio for Experiment 2 (cotton and 4N6FLOQ) and Experiment 3 (microFLOQ) samples (Kruskal-Wallis test and Dunn’s *post hoc* test, *P* < 0.05, *n* = 3)

Furthermore, regarding the samples taken using 4N6FLOQ, two out of three replicates showed contaminated STR profiles, both for the result line strips and sample pads from wet SERATEC cards. For the remaining replicate, no profile was obtained from the wet sample pad, and a non-consistent STR profile was obtained in the case of the wet result line strip from the first replicate. Comparable results were observed for samples of the dry SERATEC cards where all three replicates showed the amplification of both the Y allele of the *AMEL* gene and all the Y-STRs, pointing out the existence of contaminated STR profiles.

### Experiment 2: swab subsampling using microFLOQ swabs

All Hb tests were positive and subsampling from regular cotton swabs using microFLOQ swabs resulted in two complete STR profiles and one partial profile (44 out of 48 sequenced alleles). In contrast, three out of three complete STR profiles were obtained when subsampled from 4N6FLOQ swabs using microFLOQ. The quality of the STR profiles was assessed by calculating TPH and PHR parameters (Figure 3).

### Experiment 3: microFLOQ swabs direct sampling

It was possible to obtain full profiles from two out of three replicates of the direct sampling from the dried blood on the Falcon® tubes’ caps experiment using microFLOQ directly on the blood sample and diluting it into 60 μL of the SERATEC buffer (Figure 1C). The remaining replicate presented a non-consistent STR profile. On the other hand, all the three direct sampling replicates where 120 μL of the SERATEC buffer were used showed non-consistent STR profiles.

The quality of the STR profiles was assessed by calculating TPH and PHR parameters (Figure 3). The average TPH for the samples where 60 μL of SERATEC buffer was used equal to 2 098.57 RFUs, while the average PHR for the same samples was 0.879.

### Direct sampling vs. subsampling from cotton and 4N6FLOQ swabs

To assess the quality of the STR profiles obtained from Experiments 2 and 3 samples, two parameters were calculated: TPH and PHR. Wilcoxon tests revealed there was no significant difference between the initial swab materials, regular cotton, and 4N6FLOQ, tested for this work over either the TPH or the PHR parameters (*P* = 0.4, and *P* = 0.4, respectively) (Figure 3). Although no statistically significant difference was observed between the materials of the initial swab, the standard deviation of the TPH and PHR values is greater for regular cotton swabs than the one of 4N6FLOQ swabs.

No significant differences were observed for TPH nor for PHR between direct sampling and subsampling from the cotton, or the 4N6FLOQ swabs (*P* = 0.77, and *P* = 0.14, respectively). However, it was observed that the performance of the samples extracted from the 4N6FLOQ swabs was slightly, but not significantly (*P* = 0.4), better in terms of average peak height as well as average height ratio compared to the samples extracted with cotton and those extracted directly from the source (Figure 3).

## Discussion

In the present work, we evaluated three different strategies to integrate body fluid identification through immunochromatographic tests (SERATEC cards) with genetic profiling by direct-PCR amplification and sequencing of STRs into a single workflow. The strategies were as follows: (i) using microFLOQ swabs to subsample from SERATEC cards, both from the result test strip and from the sample pad, and both when the test was wet and when it was dry, (ii) using microFLOQ swabs to subsample from cotton and 4N6FLOQ after these were used for sampling from the source, and (iii) using microFLOQ swabs to sample directly from the source (Figure 1). These strategies were designed to maximize the use of the smallest possible sample amount, for both fluid identification and genetic profiling, considering scenarios in which the amounts of biological material are scarce. Experiment 1, by subsampling from a recently used immunochromatographic test (wet and dry). Experiment 2, by subsampling from the swabs with fresh samples from the source. Finally, Experiment 3, by direct sampling from the source using a tiny swab, microFLOQ.

**Figure 4:**
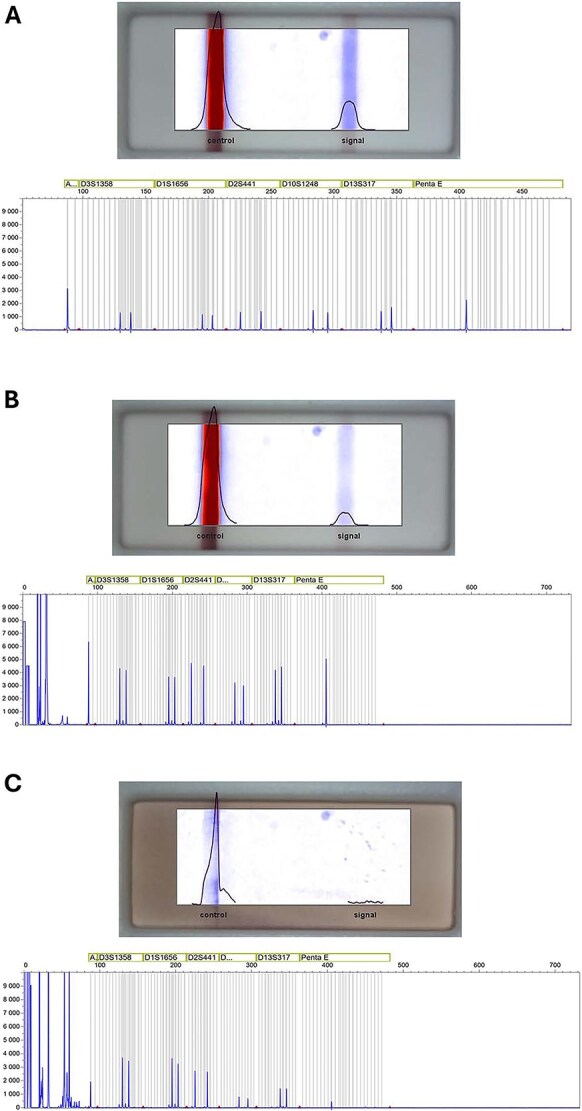
Representative pictures of the SERATEC cards analysed by the SERATEC SeraQuant reader, and their respective STR profiles. (A) High concentration sample. (B) Intermediate concentration sample. (C) Low concentration sample.

We correctly detected blood samples by immunochromatographic tests after recovering the evidence by using three types of swabs: regular cotton swabs, 4N6FLOQ and microFLOQ swabs (Supplementary Figure S1). No significant differences were found in terms of the Hb concentration among the different sampling materials after quantitation; neither a ratio nor relation between profiling success and intensity line was observed (Figure 4).

Although there have been some studies where a protein has been measured as a marker to identify human fluids indirectly by using a colorimetric quantification instrument (namely the SERATEC SeraQuant) [24], this is the first work, to the knowledge of the authors, in which Hb concentration in samples is measured indirectly from immunochromatographic tests by converting band intensity into concentration units using the SERATEC SeraQuant device.

Even though there are few studies about the performance of protocols where DNA is isolated directly from the immunochromatographic test strips [4,10,11], there is no literature to our knowledge in which subsampling is done directly from test strips and/or from swabs.

The results obtained from Experiment 1 (subsampling from the SERATEC cards) samples could be compared to the ones reported by Conte et al. [4], where the authors found no significant differences between the quantity of DNA collected from the sample pad and test strip line of SERATEC immunochromatographic PSA and amylase test strip lines. These results might show that regular amplification and direct PCR amplification are not consistent on producing genetic profiles. Additionally, the same authors reported a low rate of success for both amylase and PSA tests for STR profiles, since only one out of six replicates resulted in a full profile. No literature was found by the authors on blood tests in this context.

Subsampling using the microFLOQ swab from the SERATEC cards’ sample pad and test strip lines resulted in no complete quality STR profiles for any of the samples regardless of the swab material (cotton or 4N6FLOQ) used for the original sampling. DNA profiles obtained from most of the replicates from 4N6FLOQ swabs showed very low-intensity signals for the amplification of some STRs. However, the signals did not correspond to the donor’s profile and might correspond to contamination from the environment and/or artifacts. In contrast, profiles obtained from cotton swabs resulted in non-consistent STR profiles consisting of the amplification of several STRs with high-intensity signals that do not correspond to the donor’s profile (drop-in alleles).

In this regard, when amplifying directly using the microFLOQ swabs, the amount and the quality of DNA is unknown and thus, it cannot be optimized before amplification. For this reason, direct PCR has been reported to be more sensitive than conventional processes, potentially increasing the occurrence of background DNA amplification [25–30]. Gouveia et al. [31] reported the presence of artifacts caused by the addition of too much DNA, isolated from blood and buccal samples stored at FTA® cards, to the PCR reaction for STRs profiling.

It has also been reported that some kinds of plastics, such as polypropylene, are known to cause denaturation and multimerization of DNA [32,33]. Particularly, tandemly repeated sequences have been reported to associate with each other *in vitro* to form multimeric complexes. These complexes appear on electrophoretic gels as a series of slow-migrating bands that form a regular ladder [34–37]. This might explain the drop-in alleles and inconsistencies observed on the STR profiles from cotton and 4N6FLOQ swab samples.

**Figure 5:**
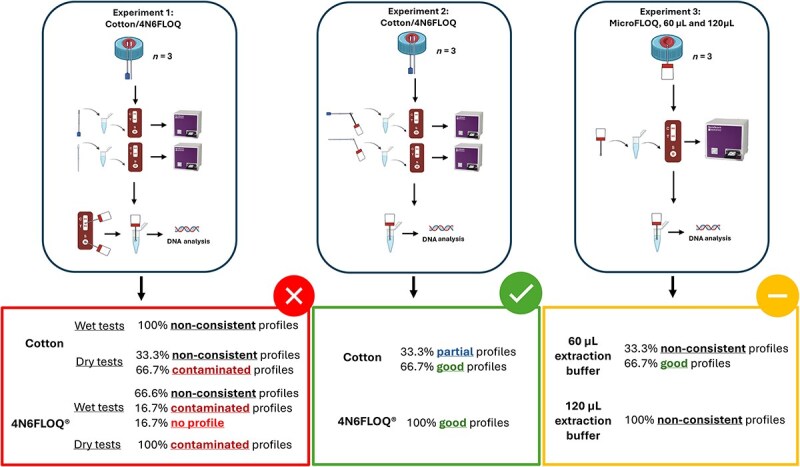
Schematic representation of the experimental design and the results of the present study for each experimental group. Swabbing from cards for DNA profiling was not good (Experiment 1), swabbing directly from the source and amplifying by direct PCR was not always successful (Experiment 3) and full profiling was successful when swabbing from both regular cotton and 4N6FLOQ swabs (Experiment 2).

In Experiment 2, subsampling from the swabs using microFLOQ swabs, two out of three replicates from the regular cotton swabs samples resulted in full profiles while the remaining replicate resulted in a partial profile made of 44/48 amplified STRs. As reported in previous works, the design of the microFLOQ swab head results in a non-absorbent core that facilitates the loss of sample due to excessive rolling, manipulation, or rubbing during sampling [15]. Variations in the STR profiles, such as the profile obtained from one of the three cotton swab samples could have been a result of minor differences in swabbing technique, differences in the amount of pressure applied, and/or because of variations in the area of the subsampled stain. On the other hand, all replicates of nylon swab subsampling resulted in complete STR profiles. The slight differences between cotton and 4N6FLOQ swabs’ STR profiles could be attributed to the subsampled substrate (swab material) as it has been reported that nylon and cotton swabs differ in their DNA collection and retrieval capabilities. Brownlow et al. [13] concluded that both nylon and cotton swabs can recover high percentages of the total amount of DNA from different samples. However, the authors reported that the nylon swab was shown to retrieve significantly more DNA compared to the cotton swab when processed using a manual extraction procedure (not automatized process). In a different study, Dadhania et al. [28] reported higher concentrations of DNA recovered from lymphocytes and buccal samples deposited on different surfaces when using 4N6FLOQ compared to regular cotton swabs.

Sampling directly from the source (Experiment 3, Figure 1C), bloodstains, using the microFLOQ, resulted in two out of three full STR profiles for the lowest volume of SERATEC buffer used (60 μL) and no full profiles for the highest volume (120 μL). Although genetic profile results were variable, all samples yielded satisfactory results for body fluid identification by immunochromatographic test cards, supporting the feasibility of this strategy for optimizing the consumption of evidence for testing. These results are in agreement with the literature, as many studies have been reported using direct PCR amplification from body fluids deposited various substrates, including natural and synthetic fabric/textile such as cotton, wool, polyester, and denim; obtaining complete STR profiles [3,38–40]. Gray et al. [38], for instance, analyzed STR profiles of samples from mock crime scenes prepared by depositing unmeasured amounts of fresh human whole blood on 45 different substrates, showing that 80% of their samples yielded a full profile (42 alleles).

Our results regarding the genetic profiles of Experiment 3 samples are not in agreement with the work of Ambers et al. [15], who reported that diluted (10%) bloodstains collected with moistened microFLOQ swabs and amplified with GlobalFiler™ Express, resulted in only seven out of 30 samples yielding full STR profiles. The authors also reported that additional diluted bloodstains (5% and 1%) obtained 28 out of 30, and 29 out 30 full STR profiles, respectively. In a similar study, Sherier et al. [41] obtained nearly complete STR profiles (97% on average, *n* = 60) using the PowerPlex Fusion 6C System by direct PCR from 10% diluted bloodstains sampled using microFLOQ swabs. However, the two works cannot be compared directly as the blood volumes and dilutions were not the same and the kit for generating the STR profiles was not the same.

## Conclusion

The present work provides evidence that an integrated workflow for body fluid identification by immunochromatographic tests and genetic profiling is feasible, either by direct sampling using microFLOQ swabs or sampling with cotton, or 4N6FLOQ swabs and subsampling with microFLOQ swabs (Figure 5).

Particularly, the immunochromatographic test proved to be useful for the proposed integrated workflow as their sensitivity was demonstrated to be sufficiently high for detecting Hb when subsampling from the swabs (cotton or 4N6FLOQ) or when sampling directly from the source and diluting the sample in SERATEC buffer. The results of this study demonstrate that microFLOQ may be a viable solution for low-quantity DNA casework scenarios by reducing the amount of sample to be used, which might be particularly useful in cases where biological evidence is scarce or of difficult access. However, further studies are required for internal validation before implementation in casework. Further considerations still need to be explored such as the time between sampling and subsampling, storage time and conditions after and before the processing step, and testing of mixed body fluids samples. The work herein serves as a proof-of-concept model for integrating body fluid identification and genetic profiling in a single workflow.

## Supplementary Material

FigS1_owaf025
