# Supplementary figures and images for: To test or not to test for body fluids: integration of body fluid identification and direct PCR in one workflow
Source: Forensic Sci Res. 2025 Sep 13;10(4):owaf025. doi: 10.1093/fsr/owaf025 (PMC12885095; doi:10.1093/fsr/owaf025)

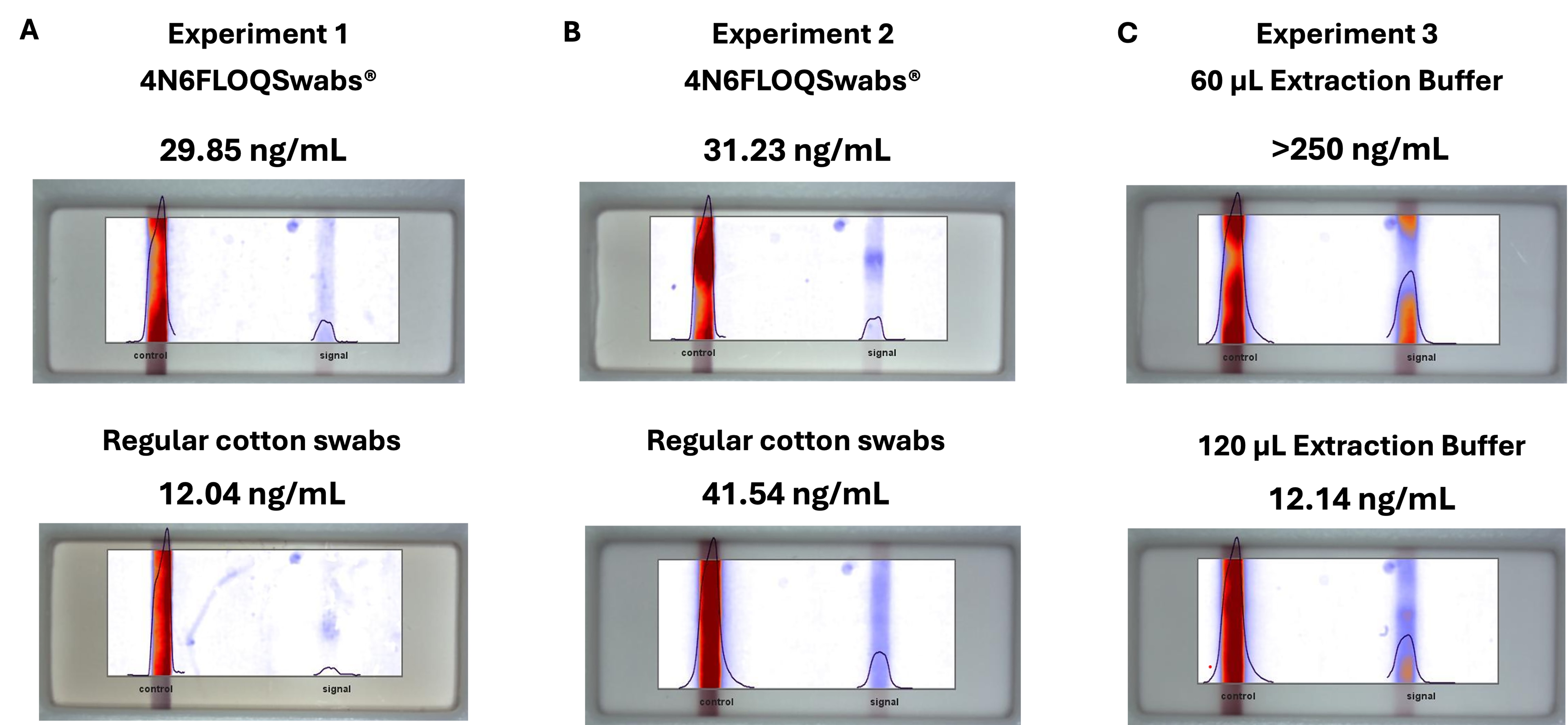

Supplement: FigS1_owaf025 [file figs1_owaf025.jpeg]
